# Preclinical Development of a Novel Zika Virus-like Particle Vaccine in Combination with Tetravalent Dengue Virus-like Particle Vaccines

**DOI:** 10.3390/vaccines12091053

**Published:** 2024-09-14

**Authors:** Dominik A. Rothen, Sudip Kumar Dutta, Pascal S. Krenger, Alessandro Pardini, Anne-Cathrine S. Vogt, Romano Josi, Ilva Lieknina, Albert D. M. E. Osterhaus, Mona O. Mohsen, Monique Vogel, Byron Martina, Kaspars Tars, Martin F. Bachmann

**Affiliations:** 1 Department of BioMedical Research, University of Bern, 3008 Bern, Switzerland; 2 Department of Immunology RIA, University Hospital Bern, 3010 Bern, Switzerland; 3 Graduate School of Cellular and Biomedical Sciences, University of Bern, 3012 Bern, Switzerland; 4 Artemis Bio-Services, 2629 JD Delft, The Netherlands; 5 Latvian Biomedical Research & Study Centre, Ratsupites iela 1, 1067 Riga, Latvia; 6 Research Center for Emerging Infections and Zoonoses, University of Veterinary Medicine Hannover, 30559 Hannover, Germany; 7 Jenner Institute, Nuffield Department of Medicine, University of Oxford, Oxford OX3 7DQ, UK

**Keywords:** virus-like particles, Zika virus, dengue virus, vaccine

## Abstract

Declared as a Public Health Emergency in 2016 by the World Health Organization (WHO), the Zika virus (ZIKV) continues to cause outbreaks that are linked to increased neurological complications. Transmitted mainly by Aedes mosquitoes, the virus is spread mostly amongst several tropical regions with the potential of territorial expansion due to environmental and ecological changes. The ZIKV envelope protein’s domain III, crucial for vaccine development due to its role in receptor binding and neutralizing antibody targeting, was integrated into sterically optimized AP205 VLPs to create an EDIII-based VLP vaccine. To increase the potential size of domains that can be accommodated by AP205, two AP205 monomers were fused into a dimer, resulting in 90 rather than 180 N-/C- termini amenable for fusion. EDIII displayed on AP205 VLPs has several immunological advantages, like a repetitive surface, a size of 20–200 nm (another PASP), and packaged bacterial RNA as adjuvants (a natural toll-like receptor 7/8 ligand). In this study, we evaluated a novel vaccine candidate for safety and immunogenicity in mice, demonstrating its ability to induce high-affinity, ZIKV-neutralizing antibodies without significant disease-enhancing properties. Due to the close genetical and structural characteristics, the same mosquito vectors, and the same ecological niche of the dengue virus and Zika virus, a vaccine covering all four Dengue viruses (DENV) serotypes as well as ZIKV would be of significant interest. We co-formulated the ZIKV vaccine with recently developed DENV vaccines based on the same AP205 VLP platform and tested the vaccine mix in a murine model. This combinatory vaccine effectively induced a strong humoral immune response and neutralized all five targeted viruses after two doses, with no significant antibody-dependent enhancement (ADE) observed. Overall, these findings highlight the potential of the AP205 VLP-based combinatory vaccine as a promising approach for providing broad protection against DENV and ZIKV infections. Further investigations and preclinical studies are required to advance this vaccine candidate toward potential use in human populations.

## 1. Introduction

Following significant outbreaks in French Polynesia in 2013 and Brazil in 2015, the Zika virus (ZIKV) continues to pose a persistent threat, inducing disease and carrying a substantial risk of neurological complications, notably Guillain–Barré syndrome and massive fetal abnormalities [1]. Spreading amongst several tropical regions with hotspots in Latin America and Africa, ZIKV caused >40,000 reported cases in 2022 in the region of the Americas alone with the highest cumulative incidence in Belize [2]. The World Health Organization (WHO) has named ZIKV as a Public Health Emergency of International Concern in 2016 [3] which brought more attention to the virus which was more unknown at that time. Especially within the epidemic in Brazil, a linkage between ZIKV infection and congenital Zika syndrome (CZS) could be detected when women were infected in the first trimester of pregnancy [4,5,6]. The debilitating effects of the Zika virus have prompted an urgent need for the creation of an effective preventive strategy. In this context, the introduction of a novel Zika vaccine has significant potential in reducing the widespread transmission and negative effects of this viral infection. As immunization of pregnant woman will be an obvious goal, well-defined recombinant, non-replicating vaccine candidates may be preferred options.

Mainly transmitted by Aedes mosquitos which acquire the virus by biting an infected person and spreading it amongst the population [7,8], the virus can also be transmitted sexually [9,10], during pregnancy [11,12,13], and through blood transfusion [14]. Despite the danger of severe complications, ZIKV infection is usually a mild, self-limiting illness with symptoms such as fever, rash, joint pain, and conjunctivitis [15]. ZIKV belongs to the Flaviviridae family and is closely related to other arboviruses such as the dengue, yellow fever, and West Nile viruses [16]. It is a single-stranded RNA virus with two lineages: an African lineage and an Asian lineage [17]. Significant phenotypic variations in the virus’ replication properties as a result of viral evolution resulted in varied regulation of the host’s innate immune response and, consequently, diverse pathogenesis [18]. ZIKV has shown the capacity to adapt and change, which has allowed it to flourish in various ecological and geographic environments. Its emergence and spread have been facilitated by factors such as climate change, globalization, and urbanization, as well as the presence of competent mosquito vectors [19].

The approximately 10.7-kilobase ZIKV genome encodes a long polyprotein that is cleaved into the capsid protein (C), precursor membrane protein (prM), envelope protein (E), and seven non-structural proteins [20,21]. The E protein interacts with host cell surface receptors to primarily mediate entry into the target cell [22,23]. The E protein is the biggest surface protein of the virus and is involved in membrane binding and fusion processes [23,24]. Like in other flaviviruses, the E protein consists of three domains. Domain I acts as a bridge between domains II and III. Since most neutralizing antibodies are directed against the receptor-binding site on domain III, this domain is the most crucial for the design of vaccines [25,26]. The fusion loop (FL) located on domain II is responsible for interacting with the host membrane during membrane fusion and is a conserved region amongst flaviviruses [27]. Due to the conserved regions, the FL is the main target for broad-reactive antibodies which contribute to antibody-mediated disease enhancement [28], owing to their good binding but poor neutralizing abilities and thus promoting viral entry into cells via FcγRs [29,30]. Therefore, many vaccines attempt to deliver domain III as the primary target.

Due to the high number of cases since the outbreaks around 2015 and the proven connection with severe neurological complications, vaccine research and development against ZIKV has intensified in recent times. To date, several ZIKV vaccines are in preclinical or human clinical trials, although none are available on the market yet [31]. Candidates are based on different vaccine platforms such as live attenuated vaccines, viral vector vaccines, and several subunit vaccines like peptide-based, protein-based, mRNA-based, DNA-based, VLP-based, and inactivated vaccines [32]. Although Zika research has made serious advances in recent years, the development of a vaccine faces several challenges. The decline in ZIKV cases makes it difficult to test vaccine efficacy in target populations, and sustained support is needed to ensure vaccine development projects are not left incomplete. Additionally, the concern of pre-existing flavivirus antibodies affecting Zika vaccine effectiveness requires further investigation. Special focus in research also needs to be put on enhancement properties amongst the flaviviruses, especially dengue in combination with Zika. Furthermore, it is vital to establish if a single vaccine can sufficiently protect vulnerable groups, such as pregnant women and children [32].

Virus-like particles (VLPs) are multiprotein supra-molecular structures that carry many characteristics of real viruses and hence present a potential vaccine platform [33]. Due to the lack of viral genome, they act as a secure template for vaccination while carrying many features of traditional vaccines [34]. It is best to deliver native antigens to the immune system in a repetitive form for B cell activation (a pathogen-associated structural pattern (PASP)) [35]; as a result, VLPs present a highly immunogenic and powerful vaccination tool that has previously been demonstrated to generate an effective immunological response [33]. Additionally, VLPs are the perfect size for direct diffusion into the lymphatic system, with sizes ranging from 20 to 200 nm (another PASP) [36]. Through several methods modifying exterior facets like chemical coupling or genetic fusion, they are able to present a specific antigen in its native form [37]. Via their bacterial expression, VLPs can deliver natural adjuvants and antigens as bacterial RNA, a natural toll-like receptor (TLR) 3, and TLR 7/8 ligands [38], enhancing the immune response additionally (a pathogen-associated molecular pattern (PAMP) [36,39,40]. The VLP used in this study, AP205, is based on an RNA bacteriophage that infects the Gram-negative bacteria Acinetobacter sp. The structure of the AP205 dimer consists of 180 copies of coat protein, arranged in a T = 3 symmetrical configuration [41]. To sterically optimize the VLP, VLP monomers were dimerized, resulting in a VLP that has 90 rather than 180 N- and C-termini, allowing fusion of larger epitopes and thus enabling their display on the VLP’s surface and eliciting robust antibody responses. One notable advantage of AP205 is its ability to accommodate the fusion of peptide epitopes, up to 55 amino acids in length, at both ends of the VLP subunit [42]. This unique characteristic allows for the display of either N-terminal or C-terminal epitopes, even if they are relatively large in size. This feature enhances the versatility and potential applications of AP205 as a promising candidate for vaccine development.

In this study, we evaluated the immunogenicity of a newly developed Zika vaccine. It is based on genetically fusing the immunocompetent EDIII domain of ZIKV to the AP205 dimer VLP. The vaccine candidate was tested in a murine model for its safety and induction of neutralizing antibodies. Since DENV and ZIKV are genetically and structurally close as well as transmitted by Aedes mosquitoes and circulate within the same ecological niche, it would be a good strategy to develop a vaccine covering all flaviviruses mentioned with a special focus on ADE. Hence, we co-formulated the ZIKV vaccine with recently generated DENV VLPs covering all four serotypes [43] and validated the immune response in mice. Using specific ADE assays, the enhancement of the induced antibodies was assessed.

In conclusion, our comprehensive investigation into the efficacy of the Zika vaccine and its combination with dengue vaccines has revealed promising results, demonstrating their abilities to induce a neutralizing response against their respective viruses; notably, the combined vaccine exhibits the potential to offer broad-spectrum neutralization without significant induction of antibody-dependent enhancement (ADE) of ZIKV and the tested DENV-2. The data from this study demonstrate that the vaccines elicit a robust neutralizing response, making the preclinical challenge in mice the logical next step for assessing the vaccines’ protective efficacy.

## 2. Materials and Methods

### 2.1. Mice

We purchased C57BL/6JOlaHsd wild-type mice from Envigo. In all in vivo studies, 8–12-week-old female mice were used. Every procedure involving animals was carried out in compliance with the Swiss Animal Act (455.109.1—5 September 2008). The Swiss Federal Veterinary Office-approved protocols were followed when treating any animal for experimentation.

### 2.2. SDS-PAGE Analysis

The SDS-PAGE analysis performed here was adapted from Krenger et al., 2023 [38]. After mixing 1 mg/mL of sample with 3 μL of reduction buffer (Thermo Scientific, Cat. 39000, Waltham, MA, USA), the mixture was heated to 95 °C for 5 min and then loaded onto a 12% SDS-PAGE with a 4% stacking gel. The gel was run at 70 V using the following buffer after a 6 μL protein ladder (Thermo Scientific, Cat. 26616) was added: SDS 0.01%, glycine 25 mM, and tris (hydroxymethyl)-aminoethane 2.5 mM. Using Azure Biosystems c300, the visible channel with auto-exposure time was used for capturing a gel photograph after protein bands were stained with InstantBlue^®^ Coomassie Protein Stain (Abcam, Cat. Ab119211, Cambridge, UK).

### 2.3. Agarose Gel Analysis

A 2% Agarose (BioConcept, 7-01P02-R, Fällanden, Switzerland) gel was loaded with a ladder (Thermo Scientific, Cat. SM0242, Waltham, MA, USA) upon mixing a 15 μL sample (1 mg/mL) and 2.5 μL loading dye (New England BioLabs, Cat. B7024S, Ipswich, MA, USA). After this, it was run in Tris-borate-EDTA (TBE) buffer at a voltage of 50 V. Gel imaging was conducted using the Azure Biosystems c300, capturing the image with the UV302 channel and utilizing auto-exposure settings.

### 2.4. AP205-ZV Vaccine Cloning, Expression, and Production

**Figure 1:**
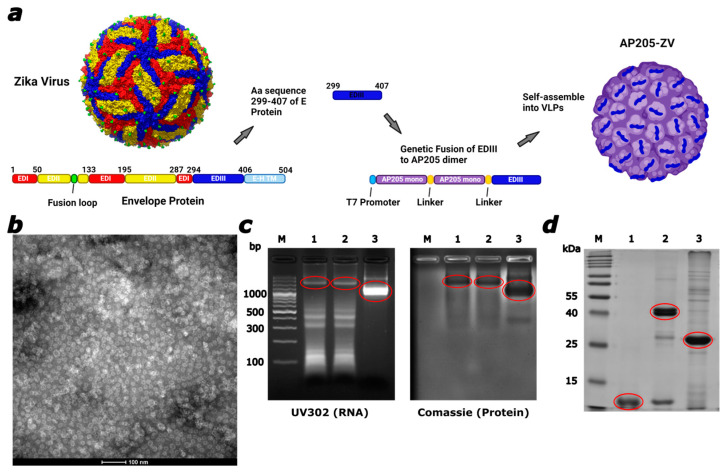
The envelope protein domain III from the Zika virus was successfully incorporated into AP205 dimer VLPs by genetic fusion. (**a**) A schematic illustration of the vaccine design. Amino acid (Aa) sequence 299–407 of the envelope protein from the Zika virus was C-terminally fused to the AP205 dimer (consisting of two AP205 monomers) and expressed in a bacterial expression system where the VLPs self-assembled. (**b**) Electron microscopy (EM) of AP205-ZV. Scale bar 100 nm. (**c**) Agarose gel analysis for visualization of packed RNA in the expressed VLPs and correlating protein staining with InstantBlue^TM^ Comassie. M. DNA Ladder, 1. AP205-ZV Batch 1, 2. AP205-ZV Batch 2, 3. AP205 dimer. Products indicated in the red circles. (**d**) 12% SDS-PAGE. M. Protein Marker, 1. ZIKV EDIII protein, 2. AP205-ZV, 3. AP205 dimer. Bands were visualized with InstantBlue^TM^ Comassie stain.

BL21 P812 *E. coli* cells were transformed with the pET-28a(+) plasmid encoding the AP205 dimer linked to the Zika virus envelope protein domain (GenBank: KY785450.1) via a GSGA linker sequence, with the EDIII domain fused at the C-terminus (Figure 1a). Post-transformation, multiple colonies were inoculated in Lysogeny broth (LB) medium and incubated overnight at 37 °C, with 100 µg/mL Kanamycin used for selection. The following day, the cultures were induced with 1 mM IPTG upon reaching an OD600 nm between 0.4 and 0.6, followed by overnight incubation at 20 °C. The cells were collected by centrifugation at 10,000× *g* for 30 min at 4 °C.

### 2.5. Protein Refolding and Purifying

For the target proteins’ purification from inclusion bodies, the cell pellet was resuspended in 10 mL of lysis buffer (PBS, 20 mM Tris, 5 mM EDTA, 5% glycerol, 0.5% Triton X-100, pH 8.0, 5 mM beta-mercaptoethanol, 1:500 lysozyme) per gram of cells. The cells were then sonicated and centrifuged at 10,000× *g* for 30 min at 4 °C, after which the supernatant was discarded. The pellet was subjected to four washes with the lysis buffer, where each wash involved resuspending the pellet, sonicating, and centrifuging at 10,000× *g* for 30 min at 4 °C. After the washes, the pellet was resuspended in inclusion body (IB) solubilization buffer (8 M urea, 50 mM Tris-HCl, 150 mM NaCl, pH 9.5) and incubated at 4 °C for 16 h on a rotating wheel. The sample was then centrifuged at 10,000× *g* for 30 min at 4 °C. The supernatant from the IB solubilization was dialyzed against refolding buffer (RB) I (2 M urea, 100 mM Na2HPO_4_, 100 mM NaH2PO_4_, 0.5 M arginine, 5 mM reduced glutathione, 0.5 mM oxidized glutathione, pH 9.5) for 24 h at 4 °C, followed by RB II (without urea) for 24–36 h at 4 °C, and finally against PBS at 4 °C. The dialyzed IB supernatant was centrifuged again at 10,000× *g* for 30 min at 4 °C, and the resulting supernatant was analyzed by SDS-PAGE. The purified proteins were further processed using a Sephacryl S-500 gel filtration column.

### 2.6. Electron Microscopy

The AP205-ZV vaccine’s physical integrity and stability have been investigated using transmission electron microscopy (Philips CM12 EM). Sample grids were glow-discharged for imaging purposes, followed by 30 s of adding 10 μL of the purified vaccine (concentration diluted to 0.7–1 mg/mL). Grids underwent a 3× ddH_2_O wash before being negatively stained for 30 s with 5 μL of 5% uranyl acetate. After removing the excess uranyl acetate, the grids were air-dried for ten minutes. Images were captured at magnifications of 84,000× and 110,000×.

### 2.7. Western Blot

A total of 5µg of protein (ZIKV EDIII, AP205-ZV, and AP205 dimer) were loaded on a 12% SDS-gel. After the run, the protein was transferred to a 0.2µm nitrocellulose membrane with the Bio Rad Trans-Blot^®^ Turbo© Transfer System (Hercules, CA, USA). The following procedure was performed with the iBindTM Flex Western System with its correlating protocol (Catalog Number: SLF2000, Invitrogen, Waltham, MA, USA, Publication No. MAN0010926) with an α-AP205 IgG (self-made) at a 1:1000 ratio as a primary antibody and secondary anti-mouse IgG Fc gamma conjugated with horseradish peroxidase (Jackson Immunoresearch Cat. No. C840T69, West Grove, PA, USA) (1:1000).

### 2.8. Binding ELISA

To test if the produced ZIKV EDIII protein and the AP205-ZV are recognized by a α- ZIKV EDIII antibody, the plates were coated with 1 µg/mL of ZIKV EDIII or AP205-ZV in PBS at a volume of 50 µL/well. The plate was incubated at 4 °C overnight. The following day, 0.01% PBS-Tween was used to wash the plates in the BioTek, 405 TS ELISA washer. The plates were incubated at room temperature (RT) for two hours on a shaker after being blocked with 100 µL/well 0.15% PBS-Casein. By flicking the plates, the blocking solution was removed. α-ZIKV-E (AA 593-699) antibody (ABIN7041072, Lot: HB13AP1802-B) was added in a volume of 50 µL/well with a concentration of 1/2000 and incubated for 1 h at RT on a shaker. Afterwards, 50 µL/well of secondary anti-mouse IgG Fcγ conjugated with horseradish peroxidase (Jackson Immunoresearch Cat. No. C840T69, West Grove, PA, USA) (1:1000) was added to the plate after it had been cleaned once more with PBS + 0.01% Tween. After another hour of incubation on a shaker, the plates were developed and cleaned, and an OD450 reading was taken (BioTek, Winooski, VT, USA).

### 2.9. Vaccination Regimen

Wild-type C57BL/6JOlaHsd mice (8–12 weeks old, Envigo) were vaccinated subcutaneously with 20 µg of either AP205-ZV in a 100 µL volume or AP205 dimer VLPs as a control, both administered without adjuvants. On day 28, the mice received an identical booster dose, and on day 56, they were terminally bled. Their sera were collected weekly via tail bleeding and separated using a Microtainer Tube (BD Biosciences, Franklin Lakes, NJ, USA).

For the follow-up experiment, the same mouse strain (wild-type C57BL/6JOlaHsd mice, 8–12 weeks old, Envigo) was used. A group of 6 mice was administered a combined dose of 20 µg (4 µg of each) of DV1-AP205, AP205~DV2, DV3-AP205, AP205-DV4, and AP205-ZV in a 100 µL volume without adjuvants. The mice received a booster with the same dose on day 28 and were terminally bled on day 49. Serum samples were collected weekly through tail bleeding and processed using a Microtainer Tube.

### 2.10. Enzyme-Linked Immunosorbent Assay (ELISA)

To determine total IgG antibody titers, Corning™ 96-Well Half-Area Plates (Fisher Scientific, Hampton, NH, USA) were coated with 1 µg/mL of ZIKV envelope protein domain III (produced in-house) in PBS (50 µL/well) and incubated overnight at 4 °C on a shaker. The following day, plates were washed with 0.01% PBS-Tween using an ELISA washer (BioTek, 405 TS, Winooski, VT, USA) and blocked with 100 µL/well of 0.15% PBS-Casein, incubating at room temperature (RT) for 2 h on a shaker. After removing the blocking solution, the serum samples were diluted in a 1:20 ratio followed by 1:3 serial dilutions, with the last row serving as a negative control. The plates were incubated for 2 h at RT on a shaker and then washed, and 50 µL/well of HRP-conjugated anti-mouse IgG Fcγ (1:1000, Jackson ImmunoResearch Cat. Nr. C840T69, West Grove, PA, USA) was added. After 1 h of incubation on a shaker, the plates were washed and developed, and the OD450 readings were taken using a BioTek microplate reader. OD50 values were calculated as the reciprocal of the 50% dilution relative to the maximal OD450. For evaluating the IgG response against DENV envelope domain III proteins, the plates were coated with 1 µg/mL of either DENV1 envelope protein domain III, DENV2 envelope protein domain III, DENV3 envelope protein domain III, or DENV4 envelope protein domain III diluted in PBS in a volume of 50 µL/well. The rest of the procedure is the same as described for the normal ELISA.

The same protocol was followed to evaluate the subclass antibody response, with the exception of using a different secondary antibody that was equivalent to the subclass: goat anti-mouse IgG1 (BD Biosciences, Cat. 559628, Franklin Lakes, NJ, USA, 1:2000 dilution), goat anti-mouse IgG2c (Southern BioTech, Cat. No. 1078-05, Birmingham, AL, USA, 1:2000 dilution), goat anti-mouse IgG2b (Invitrogen, Ref. M32407, Waltham, MA, USA, 1:2000 dilution), and goat anti-mouse IgG3 (Southern BioTech, Cat. No. 1101-05, Birmingham, AL, USA, 1:4000 dilution).

### 2.11. Avidity (ELISA)

Two sets of plates were prepared to assess the avidity of IgG antibodies. One set was treated with 50 µL/well of 7M urea in PBS + 0.05% Tween 20 and washed three times for five minutes each, while the other set was washed with PBS + 0.05% Tween 20 after serum incubation. Both sets of plates were pre-coated with 1 µg/mL of ZIKV envelope protein domain III. Between each washing cycle, the plates were cleaned using an ELISA washer with PBS + 0.01% Tween 20. The remaining steps followed the standard ELISA protocol. The avidity index was calculated by dividing the reciprocal dilution value of the urea-treated wells by the correlating value of the untreated wells.

### 2.12. Cell Culture

Eagle’s Minimum Essential Medium (EMEM) supplemented with 10% heat-inactivated fetal bovine serum (HIFBS) (Lonza Benelux BV, Breda, The Netherlands), 0.75% sodium bicarbonate (NaHCO3), 10 mM HEPES buffer, and 1% penicillin-streptomycin (Pen-Strep) (Lonza) was used to cultivate the C6/36 (CLR-1660) mosquito cell line (obtained from the American Type Culture Collection, ATCC) at 28 °C in a CO_2_-free environment.

Vero cells (ATCC^®^ CCL-81™, Manassas, VA, USA) were cultivated in complete media, consisting of Dulbecco’s modified Eagle medium (DMEM) with 10% HI-FBS (Lonza Benelux BV, Breda, The Netherlands), supplemented with 0.75% NaHCO_3_, 10 mM HEPES buffer, and 1% Pen-Strep (Lonza) at 37 °C in a humidified incubator with 5% CO_2_.

SC cells (CLR-3622), acquired from ATCC, were cultured in complete media containing RPMI 1640 supplemented with 50 µM beta-mercaptoethanol, penicillin (100 U/mL), streptomycin (100 µg/mL), and 10% HI-FBS (Gibco/ThermoFisher, Paisley, UK) at 37 °C in a humidified incubator with 5% CO_2_. All three cell lines underwent routine testing for mycoplasma using an in-house-developed RT-PCR primer and probes and consistently returned negative results.

### 2.13. Viruses

C6/36 cells were utilized to propagate dengue virus serotypes 1 (VR1856, Hawaii), 2 (VR1584, New Guinea C), and 3 (VR1265, H87)(ATCC^®^, Manassas, VA, USA). The resulting viral supernatant was then used to infect Vero cells at a multiplicity of infection (MOI) of 0.01, and the virus was harvested after 72 h. Dengue serotype 4 (VR-1490, H241) and the Zika virus (Padova 1/201)(ATCC^®^) were directly amplified in Vero cells under the same MOI conditions, with the viruses being harvested based on the observed cytopathic effects (CPEs). The harvested virus stocks were clarified via centrifugation and stored at −80 °C.

To determine the titers of the virus stocks, 10-fold serial dilutions were prepared and incubated onto Vero cells for 4 days at 37 °C in a humidified incubator with 5% CO_2_. After four days, the cells were observed under a microscope for the presence of CPEs in the case of the Zika virus. However, for dengue serotypes 1–4, the virus-infected cells were identified using an in-house-developed immunostaining method. In brief, the cells were permeabilized using 0.1% Triton-X-100 in 70% ethanol and fixed with 2.5% formalin. Then, the cells were stained using goat anti-rabbit IgG (H + L) Highly Cross-Adsorbed Secondary Antibody, Alexa Fluor Plus 488 (2 mg) (Invitrogen), and rabbit anti-flavivirus group antigen monoclonal antibody (Absolute Antibody, Oxford, UK). Thermo Fisher Scientific’s 4′,6-Diamidino-2-Phenylindole, Dihydrochloride (DAPI) was used to counterstain the nuclei of the cells. The plates were then scanned using a Cytation1V Imaging Reader (BioTek) at a 4× objective and analyzed using Gen5 software v2.1 (BioTek). The virus stock titer was calculated using the Karber formula [44].

### 2.14. Focus Reduction Neutralization Test (FRNT)

The FRNT assay was employed to assess the neutralization capacity of antibodies generated in mice immunized with different constructs, namely, DV1-AP205, AP205~DV2, DV3-AP205, AP205-DV4, AP205-ZV, and AP205dimer (used as the VLP control). The positive controls included human convalescent serum 001 against dengue virus (NR-50226)- and ZIKV-positive monkey sera, while sera from wild-type C57BL/6JOlaHsd mice served as the negative control.

The Vero cells (2 × 10^4^ cells/well) were cultivated in full media and placed in a 96-well substrate plate a day before the experiment. To deactivate complement, serum from vaccinated mice was heat-inactivated at 56 °C for 30 min. Subsequently, the serum was serially diluted two times in infection media made up of DMEM supplemented with 0.75% NaHCO_3_, 10 mM HEPES buffer, 1% Pen-Strep, and 1% HI-FBS, ranging from 1:20 to 1:160.

Subsequently, 500 TCID50 of different dengue serotypes and ZIKV were added to each well in equal volumes, followed by incubation at 37 °C for 1 h. The virus–serum mixture was then transferred to the substrate plate and further incubated for 60 min at 37 °C in 5% CO_2_. The monolayer was washed once with PBS, after which infection media were added, and the plate was again incubated at 37 °C in 5% CO_2_ for 48 h.

To quantify the percentage of infected cells, an in-house-developed immunofluorescent assay was utilized, as described above. The FRNT50 titer of each sample was determined by comparing the number of infected cells to the virus control. The FRNT50 value is determined by calculating the serum dilution at which 50% of the virus is neutralized. This was achieved by plotting the percentage of virus neutralization against the serum dilutions on a logarithmic scale and identifying the dilution that corresponded to 50% neutralization. In the FRNT assay, serum samples were tested at dilutions ranging from 1/20 to 1/160. However, in cases where the FRNT50 value exceeded the highest dilution tested, it should be noted that further dilution of serum samples would typically be required to accurately determine the endpoint titer. Due to the limited availability of serum, additional dilutions were not performed, and the FRNT50 values were extrapolated. We acknowledge that this may affect the precision of the FRNT50 estimate, and this has been addressed by presenting the percentage reduction values alongside the extrapolated FRNT50 values in the results. Each serum sample was tested in duplicate.

### 2.15. Antibody-Dependent Enhancement Test (ADE)

To assess whether the sera from vaccinated mice induced antibody-dependent enhancement (ADE) against DENV-2 and/or ZIKV, SC (CLR-3622) cells were employed. For the assay, we specifically used the serum of mice vaccinated with a mixture of DV1-AP205, AP205-DV4, and AP205-ZV. This selection was intentional and was made to avoid the inclusion of AP205~DV2, which had previously shown a strong neutralizing response and could potentially mask any ADE effects from the other vaccine components due to its dominant neutralizing antibodies. DV3-AP205 was not included in this mixture to additionally focus on the cross-protective potential of DV1-AP205 and AP205-DV4 without redundancy. It is important to note that this approach was taken for a separate study and was not originally intended for inclusion in this manuscript. As DENV3 was considered genetically closer to DENV1, its inclusion was not deemed as critical for this analysis, as the presence of less specific neutralizing antibodies theoretically increases the ADE potential. The ADE assay commenced after stimulating/differentiating SC cells (5 × 10^5^ cells/mL) with Concanavalin A (1:1000) (Merck, Germany) and Croton oil (1:20,000) (Merck) for 72 h. Upon reaching the mature macrophage-like stage, the stimulation media were withdrawn, and the cells were washed with PBS before being maintained in complete media (as described above) for an additional 48 h.

Following this, sera from immunized mice, which were serially diluted two times, were prepared, ranging from 1:20 to 1:160. Due to the limited availability of serum samples, the dilution range used in this study was restricted. While this range is typically sufficient to assess ADE based on our previous experience, we acknowledge that extending the dilution range could provide a more comprehensive evaluation and more meaningful results. For future studies, further serum dilutions will be considered to ensure the capture of the complete spectrum of potential ADE. This limitation should be taken into account when interpreting the results, and additional dilution steps would be necessary to fully assess the extent of ADE in more detailed studies. Subsequently, either dengue virus type 2/NGC strain or ZIKV Zika-Padova 1/201 was added at a multiplicity of infection (MOI) 0.1. As positive controls, mouse anti-flavivirus envelope protein IgG2a monoclonal antibody (4G2) (AbFLAVENV-4G2-200) and monkey sera previously infected with ZIKV were utilized. Sera from wild-type C57BL/6JOlaHsd mice served as the negative control.

The mixture was incubated at 37 °C for 1 h, followed by exposure to mature macrophage-like cells. After 2 h of incubation, the mixture was removed, and the cells were washed with PB and then incubated in fresh medium for 48 h. RNA was subsequently isolated from the cells’ supernatant, and pan-DENV or ZIKV real-time PCR (RT-PCR) was performed as previously described. The fold change in viral RNA levels was determined by comparing the Ct value of each sample to that of the virus control. Each serum sample was tested in duplicate.

### 2.16. Statistical Analysis

Using GraphPad PRISM 9, the data were analyzed and displayed as mean ± SEM by either one-way ANOVA or Student’s *t*-test, as indicated in the figure legends. Statistical significance was defined as *p* < 0.05 (* *p* < 0.05, ** *p* < 0.01, *** *p* < 0.001, **** *p* < 0.0001).

## 3. Results

The envelope protein domain III from the Zika virus was successfully incorporated into AP205 dimer VLPs by genetic fusion.

Since neutralizing antibodies were found to specifically target the envelope domain III of flaviviruses [25,26,45], it was employed to be fused to the AP205 virus-like particle platform to develop a new vaccine candidate against ZIKV. The ability of AP205 VLPs to fuse long and complex epitopes up to 55 amino acids either N- or C-terminally [42] makes them a potentially suitable platform for incorporating the Zika EDIII (ZV). To sterically optimize AP205 VLPs, we used a dimerized version, where two monomers are fused into a dimer. This results in 90 rather than 180 C- and N-termini, allowing the fusion of larger epitopes due to less steric hindrance [41]. The repetitive presentation of the fused antigen on the particles is efficient in the induction of humoral immune responses [37,46]. The amino acid sequence 299–407 of the ZIKV envelope protein of the Brazilian-ZKV2015 strain (GenBank: KY785450.1) was C-terminally fused to the AP205 dimer consisting of two AP205 coat proteins (CPs), displayed in a schematic illustration in Figure 1a. Through expression in E.coli, which allows subsequent easy upscaling [42], the particles can be found in inclusion bodies, refolded, and purified which results in the VLPs visualized in the electron microscopy picture in Figure 1b confirming the particle’s integrity. The yield from this expression and purification process consistently ranges between 2 and 5g per 750mL of bacterial culture. Spontaneous packing of ssRNA, serving as toll-like receptor (TLR) 3,7/8 agonist [36,38], is made possible by the assembly of the VLPs in the bacterial expression system. Antigen-presenting cells (APCs) and B cells are stimulated [47], and the most protective IgG subclasses are induced as a result [48]. The packaged ssRNA was verified in an agarose gel, seen in Figure 1c, with the confirming correlating protein staining. The produced protein has a size of around 39 kDa which is a result of fusion of the 11 kDa EDIII to the 28 kDa AP205 dimer. The observed differences in the UV302 profile and RNA content between the AP205 dimer and the modified AP205-ZV VLPs are due to the distinct production processes involved. While AP205 dimer VLPs assemble naturally in the supernatant incorporating bacterial RNA, the modified AP205-ZV VLPs undergo additional denaturation and refolding steps to ensure proper protein folding, which can affect RNA content but remains a well-controlled process. This can be seen in Figure 1d with EDIII in lane 1, AP205-ZV in lane 2, and the AP205 dimer in lane 3.

The AP205-ZV fusion product can be detected by α-AP205 and α-ZIKV EDIII monoclonal antibodies, indicating proper expression and folding of the protein.

**Figure 2:**
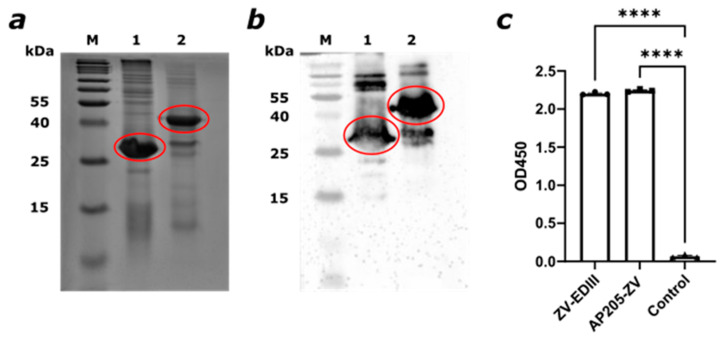
The AP205-ZV fusion product can be detected by α-AP205 and α-ZIKV EDIII monoclonal antibodies, indicating proper expression and folding of the protein. (**a**) A 12% SDS-PAGE analysis for AP205-ZV production. M. Protein Marker, 1. AP205 dimer, 2. AP205-ZV. Products indicated in the red circles. (**b**) A Western blot specific for AP205. M. Protein Marker, 1. AP205 dimer, 2. AP205-ZV. Products indicated in the red boxes. (**c**) Binding of α-ZIKV EDIII monoclonal antibody to the EDIII or AP205-ZV. EDIII and AP205-ZV coated with a concentration of 1 µg/mL. Control coated with PBS. Statistical analysis (mean ± SEM) using one-way ANOVA. One representative of two similar experiments is shown. A *p*-value < 0.05 was considered statistically significant (**** *p* < 0.0001).

To verify the proper folding of the AP205 dimer in VLPs and the displayed EDIII on its surface, a Western blot and a sandwich ELISA were performed. The AP205 protein expression was checked with a Western blot, where an α-AP205 antibody was used (Figure 2a,b). The antibody could bind the protein of interest, generating a clear signal as indicated in the figure. For confirming the expression of the ZIKV EDIII, an ELISA plate was coated with the EDIII protein alone, the AP205-ZV vaccine, and PBS as a control. α-ZIKV EDIII antibody was used as a primary antibody and an α-mouse IgG conjugated with HRP was used to develop a signal. As seen in Figure 2c, a significant signal compared to the control indicates proper folding due to the recognition of the EDIII domain by the antibody.

Immunization with AP205-ZV vaccine elicits a strong and humoral immune response of high avidity.

**Figure 3:**
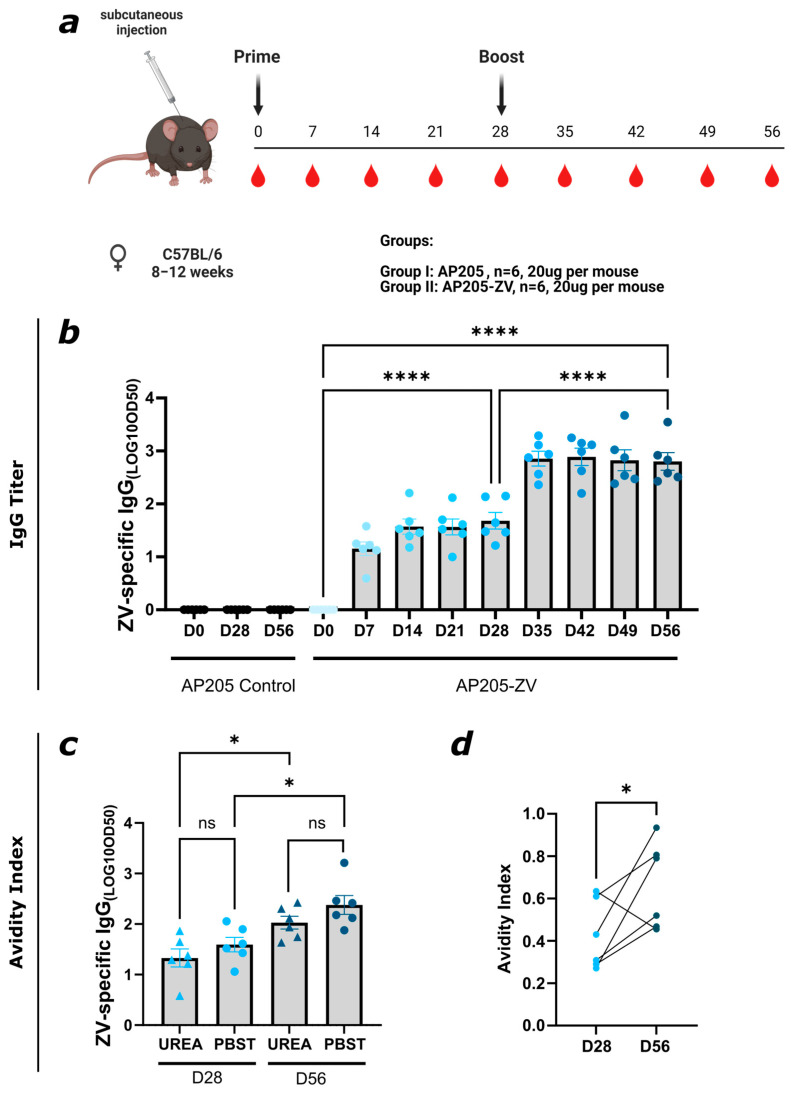
Immunization with AP205-ZV vaccine elicits a strong and humoral immune response of high avidity. (**a**) Vaccination regimen (prime on day 0 and boost on day 28, 20 μg per mouse, subcutaneous injection), bleeding time points, and vaccination groups. Created with Biorender.com. (**b**) ZV-specific IgG titer on days 0, 7, 14, 21, 28, 35, 42, 49, and 56 from mice vaccinated with AP205-ZV. For the control, days 0, 28, and 56 are shown for the group injected with AP205. Data measured by ELISA; LOG10 OD50 shown. (**c**) ZV-specific IgG titer from days 28 and 56 from mice vaccinated with AP205-ZV. After serum incubation, one plate was treated with PBS + 0.05% Tween 20 and the other plate with 7M urea in PBS + 0.5% Tween 20. LOG10 OD50 shown. (**d**) Avidity index (AI) of ZV-specific IgG titer from days 28 and 56 of the group vaccinated with AP205-ZV. Statistical analysis (mean ± SEM) using one-way ANOVA for (**b**,**c**) and paired *t*-test for (**d**). Vaccine groups, n = 6. One representative of 3 similar experiments is shown. A *p*-value < 0.05 was considered statistically significant (ns non-significant, * *p* < 0.05, **** *p* < 0.0001).

To assess the humoral immune response of the newly produced AP205-ZV, C57BL/6JOlaHsd mice were primed subcutaneously with 20 µg AP205 dimer VLPs as a control or with the AP205-ZV vaccine without adjuvant. The mice were boosted with 20 µg of the vaccine on day 28. The mice were bled on a weekly basis via their tails and were terminally bled on day 56 as illustrated in Figure 3a. Total ZV-specific IgG was measured by ELISA. After the first vaccine dose, a significant response could be detected as early as seven days after immunization. After the boost on day 28, the titer increased again significantly and reached its peak. No rise in ZV-specific IgG titer could be seen in the control group. The ZV-specific total IgG titer is visualized in Figure 3b. Since it has been shown previously that antibody avidity correlates with neutralization capacity [49], the avidity of the induced antibodies was evaluated by avidity ELISA, where plates are coated with ZIKV EDIII in duplicates and then either treated with PBST or with 7M urea to wash away low-avidity antibodies. Only the high-avidity antibodies that are still bound are indicated by the signal. In Figure 3c, the ZV-specific IgG titer on day 28 before and day 56 after the boost from the PBST- and the urea-treated plate are depicted. The response for the PBST-treated plate is slightly increased compared to the urea-treated plate on days 28 and 56. In Figure 3d, the calculated avidity index is shown. After one dose, around 40% of the antibodies are of high avidity, and after the boost, around 60% are of high avidity. Overall, these results conclude that the developed vaccine is able to induce a strong high-avidity ZV-specific IgG titer.

Vaccination with AP205-ZV induces IgG2c- and IgG2b-dominant Zika virus envelope protein domain III-specific IgG subclass response.

**Figure 4:**
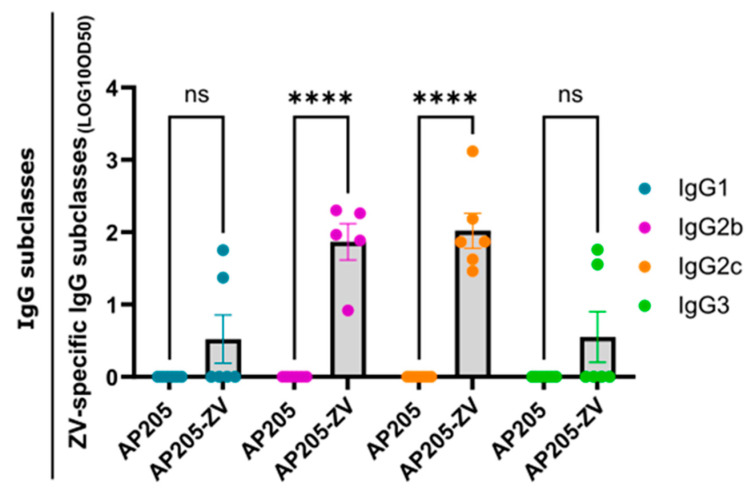
Vaccination with AP205-ZV induces IgG2c- and IgG2b-dominant Zika virus envelope protein domain III-specific IgG subclass response. ZV-specific IgG subclass titer of day 42 from mice vaccinated with AP205-ZV measured by ELISA; LOG10 OD50 shown. Statistical analysis (mean ± SEM) using Student’s *t*-test. Vaccine group, n = 6. One representative of two similar experiments is shown. A *p*-value < 0.05 was considered statistically significant (ns non-significant, **** *p* < 0.0001).

Through the packaged bacterial ssRNA inside the VLPs, TLR 7/8 is triggered, which results in subclass switching to the most protective IgG subclasses, IgG2c and IgG2b [48]. It was shown in a previous study that IgG2 were efficiently protecting from a viral challenge because the Fc-γ regions of these IgG subclasses have unique effector interaction functions [50]. Vaccination with AP205-ZV induced a significant induction of IgG2b and IgG2c, confirming previous findings. As shown in Figure 4, IgG1 and IgG3 were also induced, albeit at lower levels than IgG2b/c.

Abs induced by vaccination with AP205-ZV can recognize DENV EDIII proteins but with low avidity.

**Figure 5:**
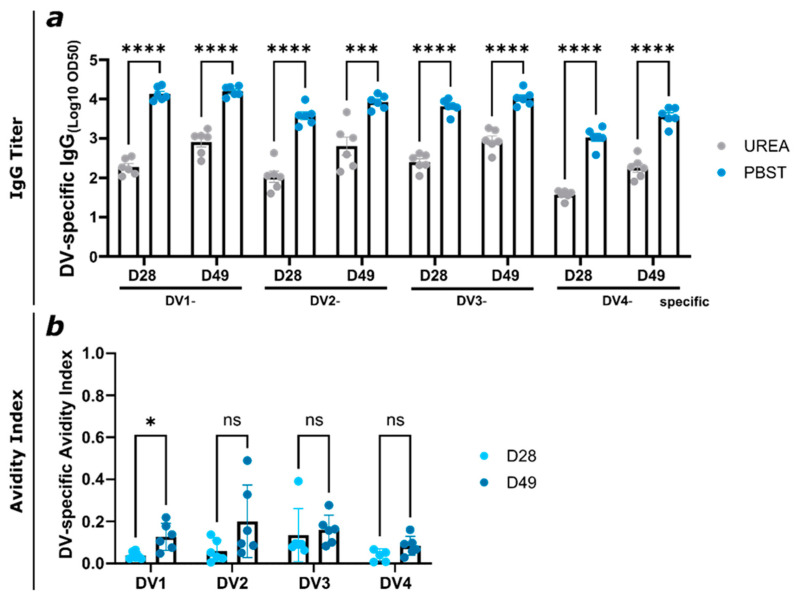
Abs induced by vaccination with AP205-ZV can recognize DENV EDIII proteins but with low avidity. (**a**) DV-specific IgG titer of days 28 and 49 from mice vaccinated with AP205-ZV measured by ELISA; LOG10 OD50 shown. After serum incubation, one plate was treated with PBS + 0.05% Tween 20 and the other plate with 7M urea in PBS + 0.05% Tween 20. (**b**) Avidity index (AI) of DV-specific induced IgG titer of days 28 and 49 of the group vaccinated with AP205-ZV. Statistical analysis (mean ± SEM) using Student’s *t*-test. Vaccine groups, n = 6. One representative of two similar experiments is shown. A *p*-value < 0.05 was considered statistically significant (ns non-significant, * *p* < 0.05, *** *p* < 0.001, **** *p* < 0.0001).

First-time DENV or ZIKV infections produce non-protective antibodies that bind to other flaviviruses but also antibodies that restrict illness upon reinfection with the same virus [51]. Non-protective antibodies may lead to antibody-dependent disease enhancement after a heterologous infection [52,53]. Since antibody avidity is the first indicator of the protective potential [49], serum induced by AP205-ZV vaccination was tested for cross-reactivity and avidity against various DENV EDIII proteins. To this end, an avidity ELISA was performed where the different DENV serotype EDIII proteins were coated. In Figure 5a, the total DV-specific IgG shows a significant difference between the PBST- and urea-treated plates. The titer was slightly higher on day 49 after the boost compared to day 28. Overall, the AP205-ZV-induced antibodies could detect the different DENV EDIII proteins strongly, although many low-avidity antibodies were washed away with the urea. This results in a low avidity index shown in Figure 5b, where for all DENV EDIII proteins, the index was below 20%. Even when the total IgG titer was high on day 28, the avidity index was very low. These results underline the literature’s findings that even if the ZIKV-induced antibodies can detect DENV EDIII proteins, they are only with low avidity, and they may therefore increase the risk for ADE in the absence of strongly neutralizing antibodies.

Vaccination against ZIKV and DENV induces strong IgG responses against EDIII proteins.

**Figure 6:**
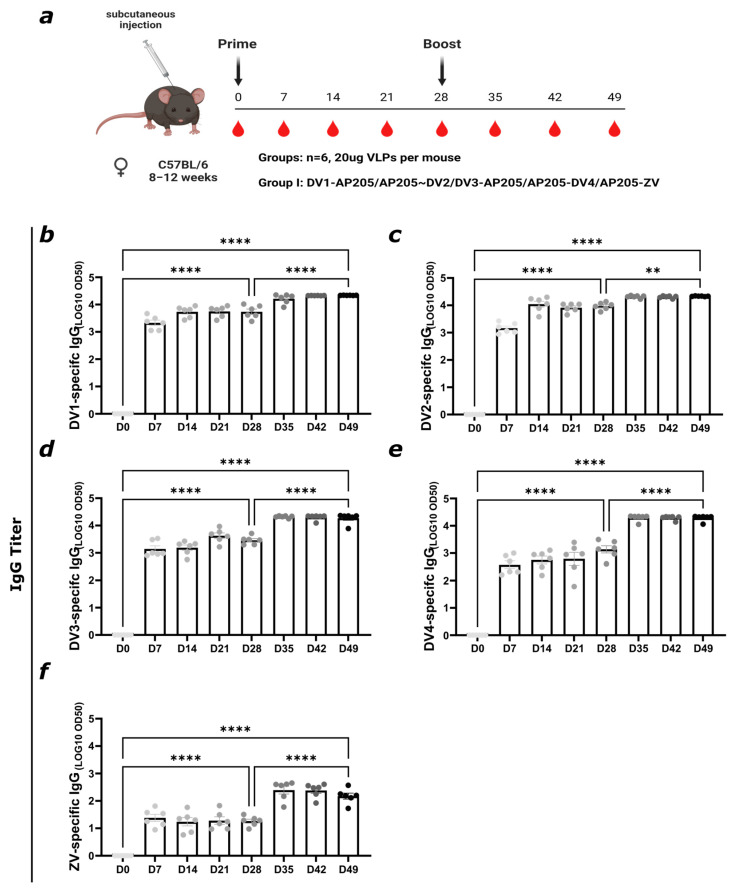
Vaccination against ZIKV and DENV induces strong IgG responses against EDIII proteins. (**a**) Vaccination regimen (prime on day 0 and boost on day 28, 20 μg of total VLPs per mouse, subcutaneous injection), bleeding time points, and vaccination group. Figure created with Biorender.com. (**b**) DV1- (**c**) DV2- (**d**) DV3-, (**e**) DV4-, and (**f**) ZV-specific IgG titer on days 0, 7, 14, 21, 28, 35, 42, and 49 measured by ELISA; LOG10 OD50 shown. Statistical analysis (mean ± SEM) using Student’s *t*-test. Group 1, n = 6. One representative of two similar experiments is shown. A *p*-value < 0.05 was considered statistically significant, ** *p* < 0.01, **** *p* < 0.0001).

Subsequently, after testing the immunogenicity of the individual ZIKV VLP, we mixed the vaccine with the previously designed DENV VLPs covering all four serotypes (see the accompanying manuscript). The total dose of the VLPs stayed at 20 µg, so 4 µg per vaccine was used for the final mix. To evaluate a vaccine covering all four DENV serotypes as well as ZIKV, the engineered VLPs were co-formulated and administrated on day 0 and day 28, as illustrated in Figure 6a. The sera were tested for detecting different EDIII proteins from ZIKV and DENV, as seen in Figure 6. The vaccine mix induced a high titer of DV-specific antibodies. The ZV-specific IgG titer was lower than in Figure 3, probably due to the lower ZV-specific dose of 4 µg. Since up to 65% of the genome from the DENV serotypes are conserved [54], the increased DV-specific titer is most likely caused by potential cross-reactive antibodies.

**Figure 7:**
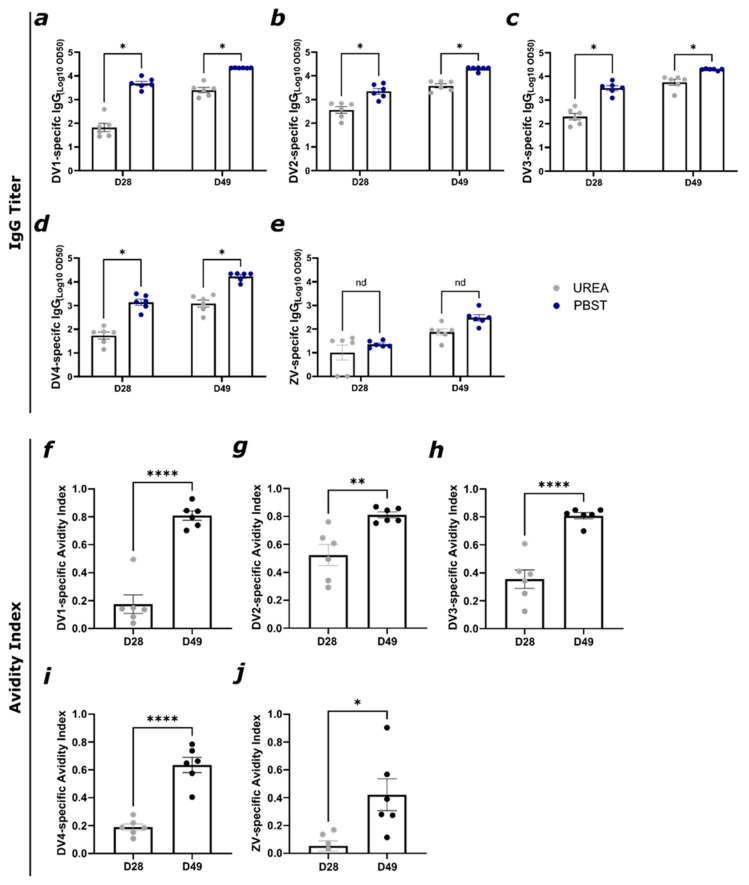
Vaccination against ZIKV and all four DENV serotypes induces high-avidity antibodies after two doses. (**a**) DV1-, (**b**) DV2-, (**c**) DV3-, (**d**) DV4-, and (**e**) ZV-specific IgG titer of days 28 and 49 from group 1 (see Figure 6a) measured by ELISA; LOG10 OD50 shown. After serum incubation, one plate was treated with PBS + 0.05% Tween 20 and the other plate with 7M urea in PBS + 0.05% Tween 20. (**f**) DV1-, (**g**) DV2-, (**h**) DV3-, (**i**) DV4-, and (**j**) ZV-specific avidity index from group 1 from day 28 and day 49. Statistical analysis (mean ± SEM) using Student’s *t*-test. Group 1, n = 6. One representative of two similar experiments is shown. A *p*-value < 0.05 was considered statistically significant (* *p* < 0.05, ** *p* < 0.01, **** *p* < 0.0001).

Figure 6b,c show similar figures for the DV1- and DV2-specific IgG titers, already reaching a titer exceeding 1:1000 seven days after being primed. Before the boost on day 28, the titer was below 4 and reached its plateau after the boost. All titers reached similar levels after the boost. For assessing the DV- and ZV-specific avidity of the induced IgG antibodies, an avidity ELISA was performed (Figure 7). In all the vaccine candidates, IgG responses were higher after the boost, and this increase was paralleled by an increase in avidity (Fig 7). The DV-specific avidity is in general higher and reaches values between 60 and 80% after the boost on day 28. The ZV-specific avidity index reaches around 40%. The higher avidity induced by the vaccine mix indicates that the induced antibodies are of higher specificity and are more likely to deliver protection through better neutralization potential [49].

AP205-ZV efficiently induces ZIKV-neutralizing antibodies.

**Figure 8:**
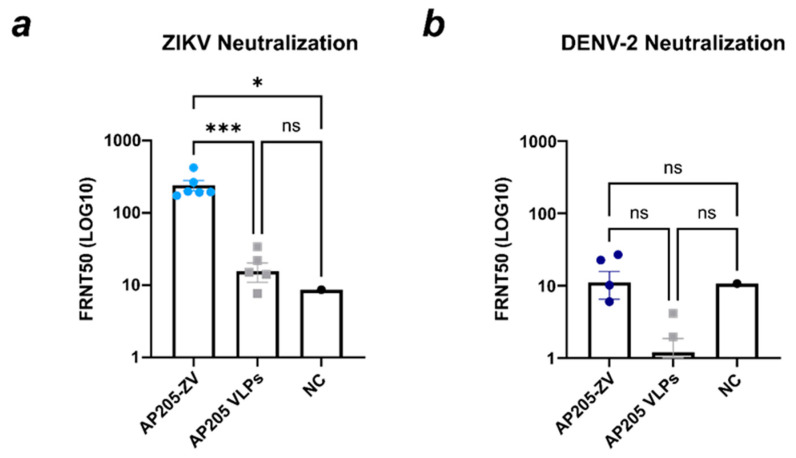
AP205-ZV efficiently induces ZIKV-neutralizing antibodies. (**a**) Foci reduction neutralization 50 (FRNT50) value from the negative control (NC), AP205-ZV-induced serum, and AP205 VLP-induced control serum. The serum was tested for ZIKV neutralization. (**b**) Foci reduction neutralization 50 (FRNT50) value from the negative control (NC), AP205-ZV-induced serum, and AP205 VLP-induced control serum. The serum was tested for DENV-2 neutralization. The serum used was from day 49. Naïve serum was used as the negative control. Statistical analysis (mean ± SEM) using one-way ANOVA. Vaccine groups, n = 6. One representative of two similar experiments is shown. A *p*-value < 0.05 was considered statistically significant (ns non-significant, * *p* < 0.05, *** *p* < 0.001).

For testing the newly developed ZIKV vaccine for its protectivity against viral infection, a VNT was performed to assess the reduction in ZIKV infection using sera from the mice vaccinated with AP205-ZV and AP205 as a control. The results showed that sera from the mice vaccinated with AP205-ZV exhibited a significantly high level of efficiency in reducing ZIKV infection, with an FRNT50 value exceeding 160 (Figure 8a). In comparison, the control serum from the mice vaccinated with AP205 VLP and the negative control (naïve serum) showed significantly lower values of 15 and 8.5, respectively. However, when the same serum samples were evaluated for their neutralization ability against DENV-2, the FRNT50 values obtained were not significantly different across all the samples and remained below 10 (Figure 8b).

Combinatory administration of AP205-ZV together with DENV VLPs induces antibodies able to neutralize all five targeted viruses.

**Figure 9:**
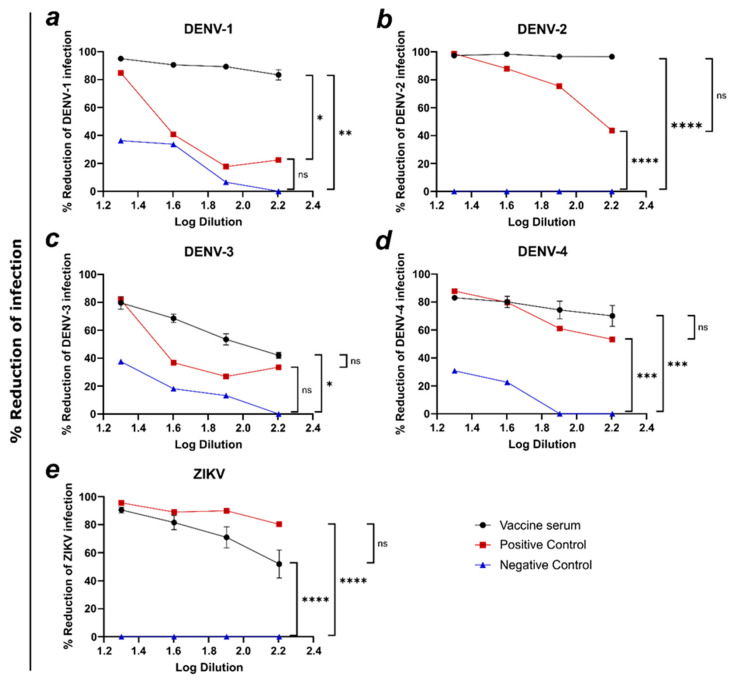
Co-formulated administration of AP205-ZV together with DENV VLPs induces antibodies able to neutralize all five targeted viruses. (**a**–**e**) Reduction in (**a**) DENV-1 infection, (**b**) DENV-2, (**c**) DENV-3, (**d**) DENV-4, (**e**) ZIKV by vaccine (DV1-AP205/AP205~DV2/DV3-AP205/AP205-DV4/AP205-ZV)-induced serum (black), positive control (red), and negative control (blue). Serum dilution is shown in Log values. Serum used from day 49. Naïve serum used as negative control. ATCC serum used as positive control. Statistical analysis (mean ± SEM) using one-way ANOVA for (**a**–**e**). Vaccine groups, n = 6. One representative of two similar experiments is shown. A *p*-value < 0.05 was considered statistically significant (ns non-significant, * *p* < 0.05, ** *p* < 0.01, *** *p* < 0.001, **** *p* < 0.0001).

To assess the neutralizing activity post-immunization with a combined vaccine (AP205-ZV and DENV VLPs), a second mouse experiment was conducted, where sera from the mice vaccinated with all four dengue VLPs and the Zika VLP were evaluated for neutralization against all the five targeted viruses. In Figure 9a, the vaccine serum reduced DENV-1 infection up to 95% at the lowest dilution, and the reduction dropped to 83%. The positive and negative controls were significantly lower (PC: *, NC: **), with the positive control reducing viral infection between 20 and 83% and the negative control between 0 and 38%. In Figure 9b, at all serum dilutions, DENV-2 infection was reduced up to almost 100% by the vaccine serum, being significantly (****) higher than the negative control. As seen in Figure 9c, DENV-3 infection was reduced by 80% at the lowest and around 40% at the highest vaccine serum dilution. The vaccine serum significantly (*) reduced DENV-3 infection compared to the negative control. In Figure 9d, the vaccine serum and positive control show a very similar reduction without a significant difference. For the vaccine serum, reduction at the lowest dilution is 81% and drops to 70% at the highest serum dilution. Both the positive control and vaccine serum reduce infection significantly (***) better compared to the negative control. For ZIKV reduction, as seen in Figure 9e, the vaccine serum is able to significantly (****) better reduce ZIKV infection compared to the negative control, with a percentage of 90% at the lowest and around 50% at the highest serum dilution.

Antibodies induced by vaccination with DV1-AP205/AP205-DV4/AP205-ZV do not enhance DENV-2, and antibodies induced by AP205-ZV do not enhance ZIKV infection.

**Figure 10:**
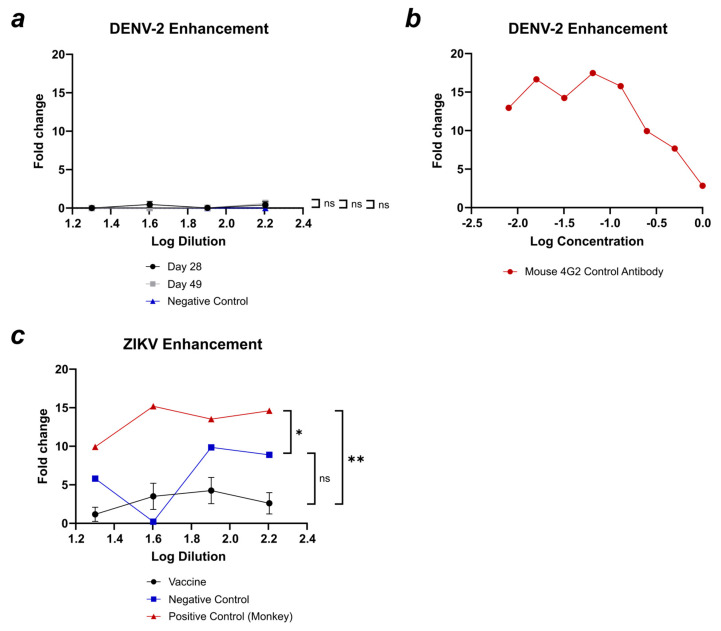
Antibodies induced by vaccination with DV1-AP205/AP205-DV4/AP205-ZV do not enhance DENV-2, and antibodies induced by AP205-ZV do not enhance ZIKV infection. (**a**) Fold change in DENV-2 infection of the serum induced by vaccination with DV1-AP205/AP205-DV4/AP205-ZV. Day 28 shown in black; day 49 shown in grey. Negative control (naïve serum) shown in blue. The serum dilution shown in Log values. (**b**) Fold change in DENV-2 infection of mouse 4G2 antibody used as a positive control. Antibody concentration shown in Log values. (**c**) Fold change in ZIKV infection of the serum induced by vaccination with AP205-ZV. Serum from day 49. Negative control (naïve serum) shown in blue; positive control (monkey serum) shown in red. The serum dilution shown in Log values. Statistical analysis (mean ± SEM) using one-way ANOVA. Vaccine group, n = 6. One representative of two similar experiments is shown. A *p*-value < 0.05 was considered statistically significant (* *p* < 0.05, ** *p* < 0.01).

Using serum from the group vaccinated with DV1-AP205/AP205-DV4/AP205-ZV, a DENV-2 enhancement assay was conducted to further evaluate the potential disease enhancement capabilities of the generated antibodies. In Figure 10a, fold change in DENV-2 enhancement for the negative control and days 28 and 49 of the induced serum is shown. All the samples did not show any increase in DENV-2 infection and varied non-significantly. A mouse 4G2 antibody was utilized as the positive control at varying concentrations, and at a concentration of 0.065 µg, it demonstrated a 17.5-fold increase in DENV-2 infection (Figure 10b). In Figure 10c, the serum induced by vaccination with AP205-ZV alone was assessed for the enhancement of ZIKV infection. For the positive control, monkey serum with a previous infection was used; this displayed a 10–15-fold change enhancement in ZIKV infection. The vaccine serum is significantly (**) lower and shows a fold change between one and three at the different serum dilutions.

## 4. Discussion

More than 3.7 million cases and more than 2000 fatalities from dengue fever have been reported from 70 nations and territories worldwide as of 23 August 2023 [55], whereas Zika has become a disease prioritized for research and development in emergency contexts as defined by the World Health Organization [56]. With these rising concerns, this study looked at a novel vaccination strategy for both the Zika virus and the four dengue serotypes.

The newly developed vaccine against ZIKV is based on a C-terminal fusion and incorporates the amino acid sequence 299–407 of the envelope protein from the Zika virus Brazil-ZKV2015 strain. The reason this sequence was used is that it plays a major role in receptor binding for viral entry [57], and it was previously shown that the EDIII domain induces mainly highly specific and neutralizing antibodies against flaviviruses [58] and is therefore known as a common target for vaccines [25,26]. The combinatory vaccination approach against DENV and ZIKV brings the risk of ADE by inducing non-neutralizing antibodies [59]. By using EDIII as a viral target, this risk can be reduced as it has been shown that cross-reactive antibodies, which are mostly non-neutralizing, target other domains and proteins on the virus, mainly the fusion loop on the envelope protein [28] or prM [60], respectively. Although EDIII contains important neutralizing epitopes, it has been suggested that it is not the most dominant epitope recognized by the immune system [61]. Factors like accessibility and changes in the conformation of E protein epitopes can affect how antibodies recognize and neutralize them [62]. So, combining EDIII with immunogenic virus-like particles might help overcome the low immunogenicity of EDIII alone, allowing us to capitalize on its crucial neutralizing epitopes.

To assess the immunogenicity of the new AP205-ZV vaccine, the humoral immune response was analyzed in a murine model for ZV-specific IgG, IgG subclasses, and avidity of the antibodies. The vaccine was able to induce a strong specific IgG response in the mice (Figure 3a), which is an essential step for the development of a new candidate since the primary target of a vaccine is to induce antibody production in B cells [63]. Furthermore, the induced IgG antibodies were of high avidity, meaning they can bind the EDIII protein strongly, as shown in Figure 3c,d. Over 60% of the antibodies were of high avidity after the boost, which may contribute to neutralization efficacy [49]. Also, through subclass switching to IgG2c and IgG2b, mediated by the packaged ssRNA in the VLPs which trigger TLR 7/8 in B cells [38,48], higher protection against viral challenge is expected since the Fc-γ regions of these IgG subclasses have unique effector interaction capabilities [50]. Indeed, a high induction of IgG2 could be observed in our data, as displayed in Figure 4.

The importance of having a vaccine that not only covers ZIKV but also the DENV serotypes is supported by the finding that a monovalent Zika vaccine that can induce cross-reactive DENV antibodies as in a natural ZIKV infection, could potentially also increase the risk of severe dengue disease following vaccination [64]. The induced cross-reactive antibodies are more likely to enhance DENV infection if they are of low avidity [65]. This constellation of cross-reactive low-avidity antibodies causes concerns for possible ADE for DENV. This phenomenon, where a high cross-reactive antibody titer can be detected upon vaccination with AP205-ZV, is likely due to the structural similarities between ZIKV and DENV EDIII, leading to cross-reactivity but with low avidity. As low-avidity antibodies are typically associated with reduced neutralization efficacy, this observation aligns with our data showing that the AP205-ZV vaccine did not significantly neutralize DENV-2, suggesting that the cross-reactive antibodies may not provide substantial immunity against DENV. These findings underscore the challenge of achieving cross-protection between ZIKV and DENV using this vaccine candidate. Hence, a pentavalent vaccine covering ZIKV and DENV could counteract the unwanted effect by induction of high-avidity and more specific antibodies which efficiently neutralize the different viruses. Therefore, we explored the humoral immune response generated by a pentavalent VLP-based vaccine formulation that included AP205-ZV alongside DENV VLPs. Notably, this formulation involved a reduction in the quantity of each VLP component compared to previous studies. Despite this reduction, the immune response elicited against the ZIKV antigen was still substantial, though the total IgG titers were slightly lower than those observed in the earlier experiments. However, the crucial aspect of this immune response lies in its functionality. The neutralizing antibody titers, assessed by a neutralization assay where the sera are mixed with live viruses and tested for its infectivity on cells, demonstrated that the pentavalent vaccine successfully induced a potent neutralizing response against all included DENV serotypes and ZIKV. These results underscore the robustness of the immune response generated by the pentavalent vaccine, highlighting that even with variations in total antibody levels, the functional quality of the antibodies remains effective in neutralizing the targeted viruses. The obtained neutralization titers for the AP205-ZV vaccine as well as for the ZIKV/DENV vaccine mix confirmed the previous findings of having developed vaccines with a good protection capacity [43] (Figure 8 and Figure 9). The AP205-ZV was able to induce a high neutralizing titer for ZIKV, supporting the data presented earlier in this study, where the vaccine was able to induce a potent humoral immune response. The serum of the AP205-ZV vaccinated mice was not able to efficiently neutralize DENV-2 in a VNT assay, validating the specificity of the engineered vaccine and again reassuring the importance of including the DENV vaccines to prevent any possible ADE. In the earlier stages of this study, it was demonstrated that antibodies induced by the ZIKV vaccine could recognize various DENV EDIII proteins, albeit with low avidity. The result from the neutralization assay aligns with the existing literature suggesting that antibodies with low avidity exhibit limited neutralization potential [49]. Furthermore, our findings corroborate previous observations indicating that while antibodies elicited by ZIKV infection can recognize DENV, their ability to effectively neutralize the virus is weak [64,65].

Furthermore, the neutralization profile of the vaccination mix containing the ZIKV and all four DENV vaccines was evaluated. Being able to reduce viral infection by 80–100% for all five tested viruses at the lowest dilution, the vaccination mix demonstrates high efficacy and highlights its promising potential for robust viral protection. Remarkably, the vaccine mix could protect the cells almost completely from viral infection, which confirms our established thesis of inducing specific neutralizing antibodies with the individual vaccines present in the vaccine mix. Furthermore, the ability to provide protection ranging from 40% to 100% at a serum dilution of 1/160 underlines the robustness of the elicited humoral immune response.

Even if the vaccine is able to induce specific neutralizing antibodies, it cannot be excluded that non-neutralizing cross-reactive antibodies were induced as well. These antibodies could potentially be harmful and lead to ADE. Especially since ZIKV and DENV are closely related within the Flavivirus family and share structural similarities, the potential for ADE needs specific research; this research should assess the vaccine for its safety for use in endemic regions, optimize the vaccine design, and predict vaccine efficacy. Several studies suggest that DENV-2 has historically been more prevalent and clinically significant in dengue-endemic regions and has been associated with more severe forms of dengue illness [66,67]. Therefore, an assay was performed to verify DENV-2 enhancement. Since in the DENV/ZIKV vaccine mix the DENV-2 neutralization titer was very high and could hinder any significant results, we tested the sera of the mice vaccinated with DV1-AP205/AP205-DV4/AP205-ZV, where the DENV-2 vaccine was missing (see Supplementary Figure S1). However, no enhancement was seen compared to the positive control and negative control, which further states the safety profile of the vaccine administrated in a co-formulated manner. To verify that the ZIKV vaccine alone does not induce ZIKV-enhancing antibodies, the serum was tested as well, and no enhancement was detected. The potential risk of ADE remains a critical concern in the development of vaccines against Flaviviruses. ADE can occur not only through the induction of non-neutralizing antibodies but also in scenarios where the concentration of neutralizing antibodies is insufficient for effectively controlling viral replication. Our results with the pentavalent VLP-based vaccine mix, which includes components targeting both DENV and ZIKV, showed the induction of neutralizing antibody titers. However, achieving a more balanced and robust humoral immune response is crucial to mitigating the risk of ADE.

Given the results, which indicate that while neutralizing titers were achieved, further strategies may be necessary for enhancing and balancing the immune response more effectively. This may include optimizing vaccine doses, refining the VLP formulations, or considering adjuvants that could boost the overall immunogenicity of the vaccine. Moving forward, our focus will be on addressing these challenges to enhance the vaccine’s efficacy and safety profile.

## 5. Conclusions

In this study, we collectively developed a novel VLP-based Zika virus (ZIKV) vaccine, evaluating its immunogenicity, neutralization capacity, and antibody-dependent enhancement (ADE) potential. Additionally, we administered and analyzed this vaccine alongside DENV VLP vaccines, exploring its potential as a multitarget vaccine against both the dengue virus (DENV) and ZIKV. Our ZIKV vaccine candidate, AP205-ZV, efficiently elicited a robust humoral immune response and demonstrated effective ZIKV neutralization without inducing ADE for ZIKV. When combined with DENV vaccines covering DENV1-4, our vaccine-induced highly specific, high-avidity antibodies capable of efficiently neutralizing ZIKV and all four DENV serotypes while avoiding disease enhancement for DENV-2. These attributes underscore the significant potential of our novel vaccine. Given the ongoing global threat posed by ZIKV and DENV, characterized by numerous infections and fatalities in endemic regions, further investigation of this combinatory vaccination approach is warranted. To further evaluate the vaccine’s protective efficacy, the next step will involve conducting a murine challenge model study.

## Data Availability

Data are contained within this article and Supplementary Materials.

## Supplementary Materials

The following supporting information can be downloaded at https://www.mdpi.com/article/10.3390/vaccines12091053/s1, Supplementary Figure S1: Vaccination with DV1-AP205/AP205-DV4/AP205-ZV induces strong IgG responses against EDIII proteins. Supplementary Figure S2: Stability of AP205-ZV after 6 months of storage at 4 °C. Supplementary Figure S3: Fluorescence microscopy images of controls on cells for neutralization assays.
