# Supplementary material for: Preclinical Development of a Novel Zika Virus-like Particle Vaccine in Combination with Tetravalent Dengue Virus-like Particle Vaccines
Source: Vaccines (Basel). 2024 Sep 14;12(9):1053. doi: 10.3390/vaccines12091053 (PMC11435730; doi:10.3390/vaccines12091053)
Supplement: Supplementary file 1 [file vaccines-12-01053-s001.zip › vaccines-3145898-supplementary.pdf]

Supplementary Materials

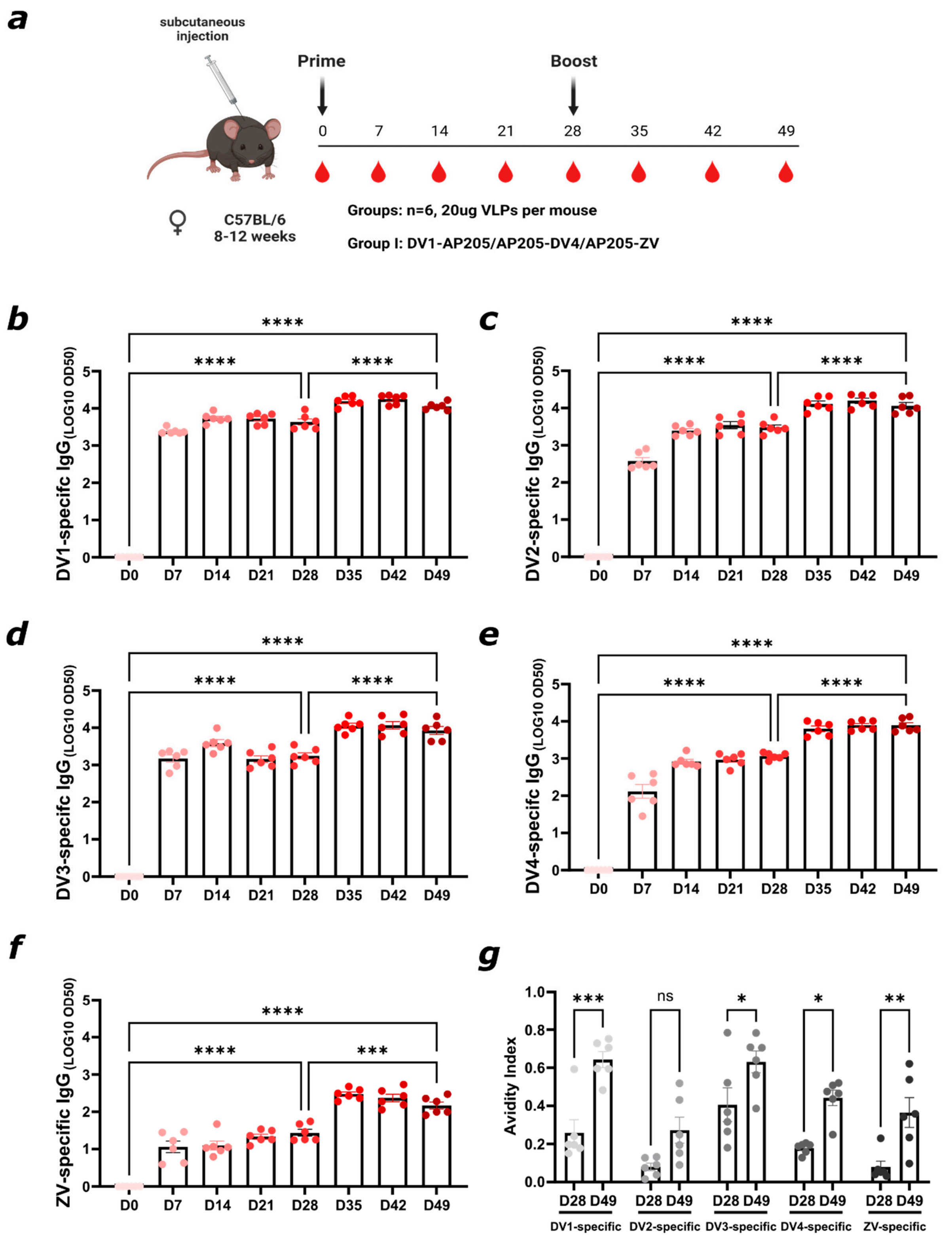

Supplementary Figure S1. Vaccination with DV1-AP205/AP205-DV4/AP205-ZV induces strong IgG responses against EDIII proteins. ***a*** Vaccination regimen (prime on day 0 and boost on day 28, 20ug of total VLPs per mouse, subcutaneous injection), bleeding time points and vaccination group. Figure created with Biorender.com. ***b*** DV1- ***c*** DV2- ***d*** DV3- ***e*** DV4- ***f*** ZV-specific IgG titer on days 0, 7, 14, 21,

28, 35, 42 and 49 measured by ELISA, LOG_{10} OD_{50} shown. ***g*** EDIII-specific Avidity Index from group 1 from day 28 and day 49. Statistical analysis (mean ± SEM) using *Student`s t-test* for ***a–f*** and Two-way ANOVA for ***g***. Group 1 n = 6. One representative of 2 similar experiments is shown. The value of $p < 0.05$ was considered statistically significant (*p < 0.05, **p < 0.01, ***p < 0.001, ****p < 0.0001).

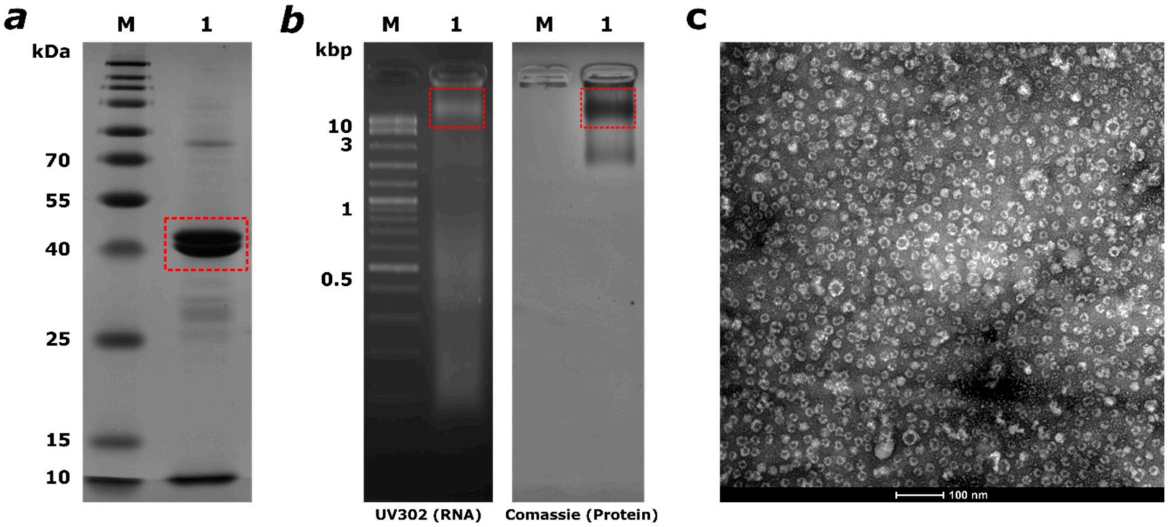

Supplementary Figure S2. Stability of AP205-ZV after 6 months storage at 4 °C. ***a*** 12% SDS-PAGE for AP205-ZV after 6 months storage at 4°C. M. Protein marker, 1. AP205-ZV. AP205-ZV indicated in the red box. Bands were visualized with InstantBlue™ Comassie stain. ***b*** Agarose gel analysis to visualize the packed RNA in the VLPs and the correlating protein staining with Comassie. M. DNA Ladder, 1. AP205-ZV. AP205-ZV indicated in the red boxes. ***c*** Electron microscopy (EM) of AP205-ZV. Scale bar 200nm.

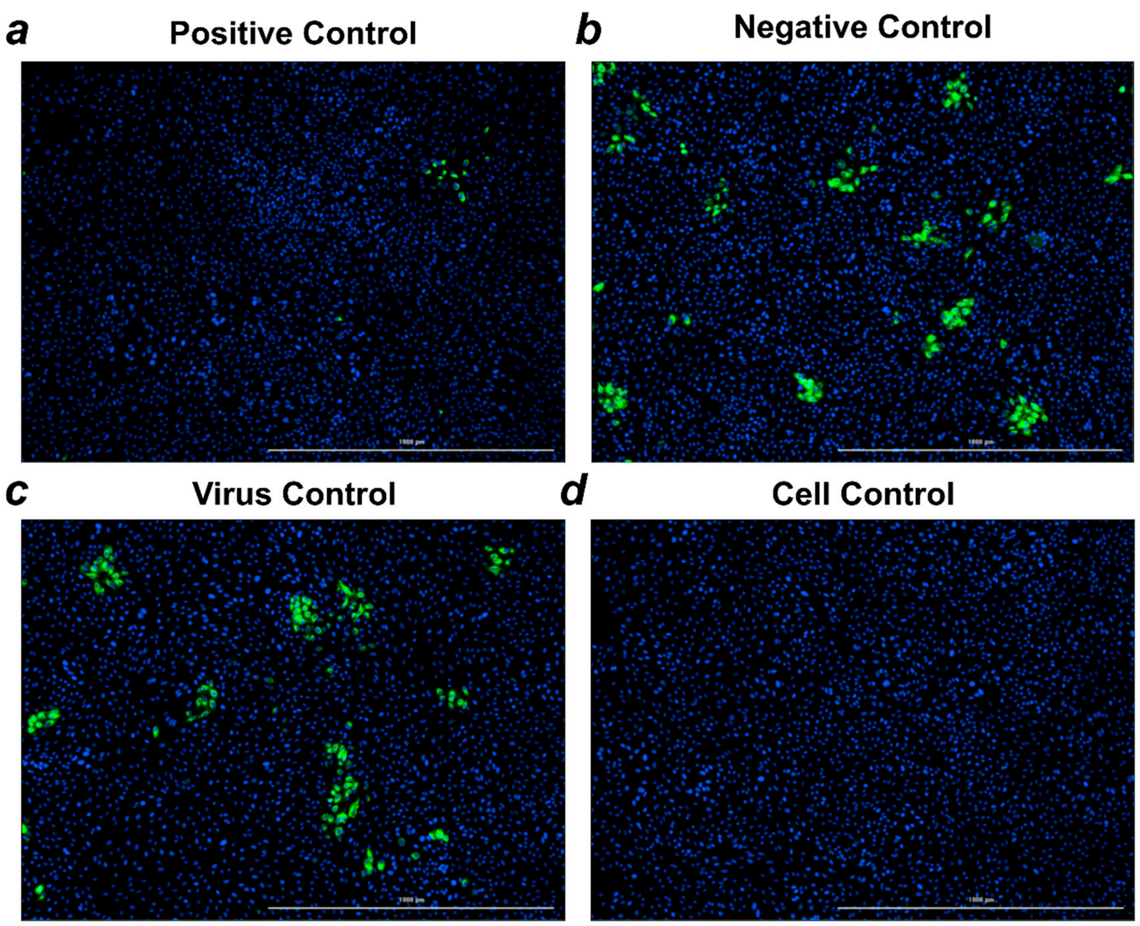

Supplementary Figure S3. Fluorescence Microscopy images of controls on Cells for the Neutralization Assays. ***a*** Positive Control. ***b*** Negative Control. ***c*** Virus Control. ***d*** Cell Control. DAPI nucleic acid stain is depicted in blue, infected cells are depicted in green. Scale bar 1000 µm.
